# Obesity and statins are both independent predictors of enhanced coronary arteriolar dilation in patients undergoing heart surgery

**DOI:** 10.1186/1749-8090-8-117

**Published:** 2013-04-30

**Authors:** James Cassuto, Attila Feher, Ling Lan, Vijay S Patel, Vinayak Kamath, Daniel C Anthony, Zsolt Bagi

**Affiliations:** 1 Vascular Biology Center, Medical College of Georgia, Georgia Regents University, Augusta, GA, USA; 2 Department of Biostatistics and Epidemiology, Medical College of Georgia, Georgia Regents University, Augusta, GA, USA; 3 Department of Surgery, Medical College of Georgia, Georgia Regents University, Augusta, GA, USA; 4 Department of Pharmacology, University of Oxford, Mansfield Road, Oxford OX1 3QT, UK

**Keywords:** Obesity paradox, Microcirculation, Coronary arteriole, Statin

## Abstract

**Background:**

A paradoxical inverse relationship between body mass index, morbidity and mortality in patients with ischemic heart disease has been noted; but the underlying mechanisms remain unclear. Given that coronary resistance arteries are the primary regulators of myocardial blood flow, we examined the effects of obesity and medication on dilator function in coronary microvessels.

**Methods:**

Bradykinin-induced coronary dilation was assessed by videomicroscopy in *ex vivo* coronary arterioles obtained from 64 consecutive patients undergoing heart surgery. Multi-variable linear regression and logistic regression were used to investigate the effects of obesity (BMI ≥ 30 kg/M^2^) and the influences of medications on vessel responses.

**Results:**

In isolated, pressurized (80 mmHg) coronary arterioles of obese and non-obese patient the active (73±4 vs. 79±13 μm) and passive (111 ± 5.5 vs. 118 ± 5.0 μm) diameters were similar. Bradykinin elicited substantial dilation in coronary arterioles, with a similar magnitude in obese and non-obese patients (to 10^-8^ M: 55 ± 5% vs. 46 ± 5%, *P* = 0.20), but with significantly enhanced sensitivity in obesity (EC50: 8.2x10^-9^ M vs. 1.9x10^-8^ M, respectively, *P* = 0.03). When adjusted for other risk factors and medications, obesity and statins were determined to be the only positive predictors of enhanced dilation, as assessed with multiple regression analysis. Moreover, obese patients with or without statin exhibited significantly increased coronary dilation to bradykinin, when compared to non-obese patients without statin therapy.

**Conclusions:**

Obesity and statin therapy are independently associated with an enhanced dilator function of coronary arterioles in patients undergoing heart surgery, which may offer a potential mechanism for the better cardiovascular outcome described earlier as the obesity paradox.

## Background

Obesity is an important determinant of ischemic heart disease (IHD) [1]. The adverse effects of obesity on the cardiovascular system include the development of hypertension, left ventricular hypertrophy, heart failure and coronary artery disease [2,3]. Given the adverse effects of obesity on the coronary circulation, one might predict inadequate coronary perfusion of the myocardium. In support of this outcome, studies conducted in postmenopausal women have shown that obesity is associated with reduced myocardial blood flow and reduced coronary flow reserve [4,5]. Schindler et al. have demonstrated no difference in myocardial blood flow between obese and lean subjects under resting conditions, but increases in myocardial demand following simulation with cold pressor and pharmacologic tests resulted in significantly reduced blood flow in the obese population [6]. In contrast to these studies, the Multi-Ethnic Study of Atherosclerosis (MESA), which involved 222 men and women, demonstrated that neither the resting myocardial blood flow nor adenosine-induced hyperemic flow were correlated with obesity in asymptomatic patients [7].

Despite the anticipated deleterious effects of obesity on cardiovascular outcomes, several studies have revealed that in specific clinical settings, obese patients with advanced IHD exhibit reduced morbidity and mortality compared to disease-matched lean patients. This has become known as the ‘obesity paradox.’ For example, in an analysis of the Acute Decompensated Heart Failure National Registry, Fonarrow et al. demonstrated that for every five unit increase in body mass index (BMI), the risk adjusted mortality was 10% lower [8]. Kaplan-Meier analysis, following percutaneous coronary intervention with new generation drug eluting stents, revealed improved survival in obese patients with metabolic syndrome compared to those without metabolic syndrome (96.59% vs. 91.75%, P = 0.04) [9]. Using contrast enhanced MRI, Pingitore et al. demonstrated significantly reduced infarct size following myocardial infarction in obese (11 ± 4% of left ventricular myocardium) compared to normal body weight patients (16 ± 9% left ventricular myocardium, *P* = 0.03) [10]. The underlying mechanisms responsible for these observations remain obscure.

In a previous study, we observed enhanced dilator function of coronary resistance arteries to bradykinin in a subset of obese patients with hypertension who underwent heart surgery [11]. We hypothesized that coronary arterioles may adapt to the hemodynamic changes in obesity in order to meet the increased metabolic needs in the diseased heart [12,13]. However, due to the array of medications prescribed to obese patients for the management of hypertension and dyslipidemia, it is also plausible that the perceived enhancement in coronary dilator function in obesity is a consequence of pharmacologic agents having beneficial effects on coronary dilator response. Thus, in the present study, we set out to elucidate the independent impact of obesity and also medications adjusted for other co-morbidities on the dilatory function of coronary arterioles. Our goal is to ascertain if vascular adaptations to obesity exist in the human coronary microcirculation, independent of the effect of pharmacologic agents. We evaluated vasodilation in response to the endothelium-dependent agonist, bradykinin, in isolated coronary arterioles obtained from patients who underwent heart surgery. Detailed statistical analyses were performed to detect the effects of obesity and medications and the influence of co-morbid conditions, such as hypertension and diabetes mellitus on these responses.

## Methods

All protocols were approved by the Institutional Review Board at Georgia Regents University, Augusta, GA. All tissue samples included in the study were surgical surplus. In order to protect patients’ privacy, all tissue samples and demographic data were de-identified.

### Assessment of bradykinin-induced dilation in *ex vivo* coronary arterioles

Twenty-eight obese (BMI ≥ 30 Kg/M^2^) and thirty-six non-obese (BMI ≤ 30 Kg/M^2^) consecutive patients who underwent heart surgery were included in this study. With the use of microsurgery instruments and a dissecting microscope, coronary arterioles were dissected (approximately 100 μm in diameter and 1.5 mm in length) from right atrial appendages acquired at the time of heart surgery. After transferring the isolated arterioles to an organ chamber, vessels were cannulated at both ends with glass micropipettes that were attached by silicone tubing to hydrostatic pressure reservoirs to set intraluminal pressure to 80 mmHg. The organ chamber was continuously perfused with physiologic salt solution at a temperature of 37°C. The internal diameter of the isolated coronary arterioles was assessed by video microscopy. Changes in diameter were continuously recorded with a videocaliper (Texas Instruments) and analyzed with AcqKnowledge data-acquisition software (Biopac Systems).

In the experimental protocols, coronary arterioles were allowed to develop a spontaneous basal tone without the use of any pre-constrictor agent. Then, changes in diameter in response to increasing concentrations of bradykinin [10^-10^ - 10^-7^ M] were measured. This approach is an established method for assessing endothelial-dependent dilator function of human coronary arterioles [11,14]. Diameter changes were expressed as percent changes in arteriolar dilation (Δ diameter in response to bradykinin/passive diameter, obtained in calcium free physiologic salt solution). The half maximal effective concentration (EC50) of bradykinin was modeled and calculated for non-obese, obese, and the entire study population using a sigmoidal dose response model (variable slope).

### Statistical analyses

All patients included in the study had complete data profiles. All statistical tests were performed using SPSS version 18 (PASW) with a significance level of 0.05 unless otherwise noted. Descriptive analyses were conducted on patient characteristics by obesity status. T-tests and Chi-squared tests were used to compare continuous (i.e.: age) and categorical (i.e.: gender, co-morbidities) variables, respectively, between obese and non-obese populations. Univariate analyses were conducted to identify possible important risk factors between BMI and arteriole dilation. To delineate the associations between obesity and other risk factors for the magnitude of enhanced dilation, two-way ANOVA was used to evaluate interactions between obesity and co-morbid conditions (diabetes, hypertension, coronary artery disease, hypercholesterolemia), and between obesity and major medications (ACE inhibitors, angiotensin receptor blockers, aspirin, statins, insulin, anti-diabetics, beta blockers, diuretics, calcium channel blockers, H^+^ blockers, nitrates) patients were taking at the time of surgery on coronary arteriole dilations to bradykinin. These tests were performed to limit the degree of confounding in multivariable models.

Multivariable linear and logistic regressions were used to evaluate the impact of obesity on vasodilator function (vessel responses to bradykinin) controlling for the effects of patient medications. Backward model selection procedures were used to find the best fitting multivariable and logistic regression models. Logistic model predictions were evaluated with Hosmer and Lemeshow tests for goodness of fit. The initial multivariable models included all marginally statistically significant variables (p < 0.10) selected by Chi-squared tests in preliminary univariate analyses and possible interactions were also examined before finalization of the model. The possible risk factors included in the initial models were obesity (BMI ≥ 30), age, gender, ace inhibitors (ACE-I), angiotensin receptor blockers (ARB), statins, insulin, oral anti-diabetics, diuretics, and calcium channel blockers. The final multiple regression model included obesity and statins. The final multivariable logistic regression models included obesity, statins, ARB, and ACE-I.

G*Power 3.1.2 was used for post-hoc power analyses [15]. In the power analysis for the 2-way ANOVA the *P* value was set to 0.05 and numerator degrees of freedom set to 2. For Chi-squared tests the *P* value was set to 0.05 and the degrees of freedom set to 1. Post Hoc power analysis of the 2-way ANOVA demonstrated a power of 0.40 to detect small (f = 0.25) and a power of 0.81 to detect large (f = 0.40) interactions. Post Hoc power analysis of Chi-squared tests demonstrated a power of 0.13 to detect small (w = 0.10) and a power of 0.98 to detect large (w = 0.50) differences between the groups (obese and non-obese patients). The F test was used to compare the bradykinin EC50 between obese and non-obese patients with GraphPad Prism 5 software.

## Results

### Patient characteristics

Table 1 provides details of the demographic data. The average BMI for the obese population was 33.4 kg/m^2^ (standard deviation (SD) = 3.8) compared to 26.3 kg/m^2^ (SD = 3.1) for the non-obese subjects. BMI was normally distributed across the entire population (mean ± SD=29.4±4.9 kg/m^2^). The distribution of co-morbid conditions between the two populations was similar based on Chi-squared test. Compared to obese patients, more non-obese patients were on ACE inhibitors prior to surgery (28.6%, vs. 55.6%, *P*=0.040), whereas more obese patients were on beta-blockers (58.3% vs. 89.3%, P=0.009) and proton pump inhibitors (39.3% vs. 16.7%, P=0.049). Obese patients were more likely than non-obese patients to undergo surgery for coronary artery bypass procedures (89.3% vs. 69.4%, P=0.049), while more non-obese patients underwent aortic valve replacement surgery (14.3% vs. 50.0%, P=0.003).

**Table 1:** Patient demographics

	**BMI ≥ 30**	**BMI < 30**	**P value**
**Number (n)**	28	36	
**Body mass index - Kg/M**^**2**^**(mean ± SD)**	33.4 ± 3.8	26.3 ± 3.1	< 0.001
**Age - years (mean ± SD)**	69.6 ± 6.8	65.5 ± 15.5	0.193
**Male (%)**	20 (71.4)	30 (83.3)	0.278
**Coronary response**			
**Passive diameter - μm (mean ± SEM)**	111.3 ± 5.5	118.1 ± 5.0	0.369
**Bradykinin response [E**^**-8**^**M] -% dilation (mean ± SEM)**	54.6 ± 4.7	45.9 ± 4.7	0.199
**Underlying disease**			
**Diabetes mellitus (%)**	13 (46.4)	17 (47.2)	0.950
**Hypertension (%)**	25 (89.3)	31 (86.1)	0.672
**Coronary artery disease (%)**	13 (46.4)	18 (50.0)	0.886
**Hypercholesterolemia (%)**	22 (78.6)	26 (72.2)	0.691
**Surgery**			
**Coronary artery bypass graft (%)**	25 (89.3)	25 (69.4)	0.049
**Aortic valve replacement (%)**	4 (14.3)	18 (50.0)	0.003
**Mitral valve replacement (%)**	1 (2.8)	0 (0.0)	0.374
**Medications**			
**ACE inhibitor (%)**	8 (28.6)	20 (55.6)	0.040
**Angiotensin receptor blocker (%)**	13 (46.4)	10 (27.8)	0.144
**Aspirin (%)**	23 (82.1)	27 (75.0)	0.456
**Statin (%)**	15 (53.6)	26 (72.2)	0.087
**Insulin (%)**	6 (21.4)	7 (19.4)	0.889
**Anti-diabetics (%)**	6 (21.4)	7 (19.4)	0.889
**Beta blocker (%)**	25 (89.3)	21 (58.3)	0.009
**Diuretic (%)**	6 (21.4)	15 (41.7)	0.073
**Calcium channel blocker (%)**	13 (46.4)	16 (44.4)	0.955
**H+ blocker (%)**	11 (39.3)	6 (16.7)	0.049
**Nitrate (%)**	8 (28.6)	4 (11.1)	0.085

All data, unless specified otherwise, represent number (n) and frequencies (%) of distribution in parentheses. *P* values for comparison between obese (BMI ≥ 30) and non-obese patients were calculated using Chi-squared test or t-test for categorical or continuous variables respectively.

### Coronary arteriolar dilation to bradykinin in obese and non-obese patients

**Figure 1:**
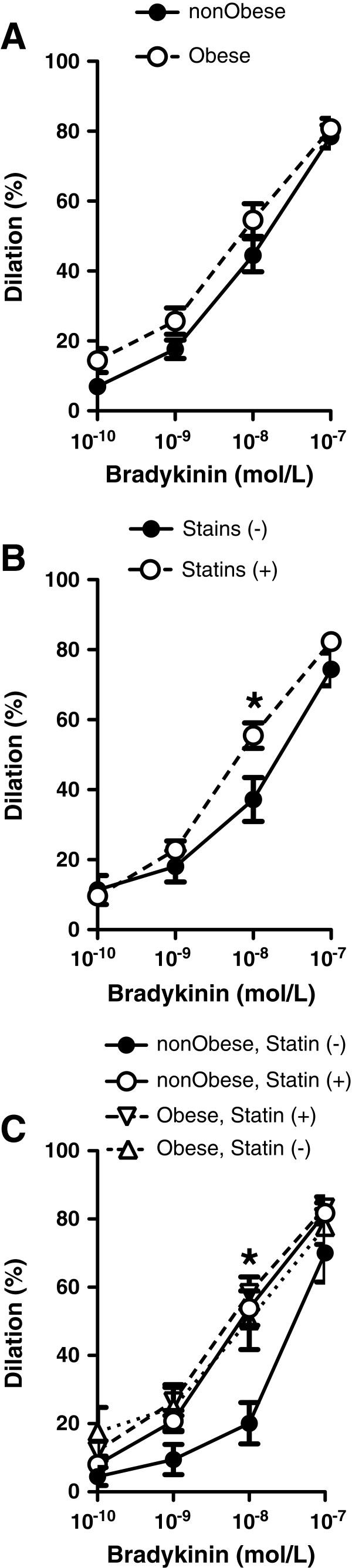
**Bradykinin induced dilations of coronary arterioles.** Dilations (%) in response to cumulative concentrations of bradykinin in isolated coronary arterioles obtained from non-obese and obese patients (**A**). Coronary dilation to bradykinin stratified by patient use of statins (**B**) and also stratified by both statin and obesity status (**C**).

The active diameter and spontaneously developed myogenic tone of isolated, pressurized (80 mmHg) coronary arterioles was similar in obese (73±4 μm, 32.6±3% myogenic tone) and non-obese patients (79±13 μm, 30.0±6% myogenic tone) (*P*=0.175 and *P*=0.465, respectively). Also, there was no significant difference between the passive coronary diameters in obese and non-obese patients (111 ± 5.5 μm vs. 118 ± 5.0 μm, respectively, P=0.369). Bradykinin elicited a substantial, dose-dependent dilation of coronary arterioles in both groups (Figure 1A). The EC50 for bradykinin-induced dilations was 1.3x10^-8^ M (95% CI = 3.9x10^-9^ to 4.1x10^-8^) for the entire study population, 8.2x10^-9^ M (95% CI = 2.6x10^-9^ to 2.5x10^-8^) for the obese patients, and 1.9x10^-8^ M (95% CI = 1.7x10^-9^ to 2.2x10^-7^) for the non-obese population (*P* = 0.032 for obese vs. non-obese). These data indicated a significantly increased sensitivity to bradykinin of coronary arterioles in obese patients. As the EC50 for the entire population approximated to 10^-8^ M, this bradykinin concentration was used to assess the vasodilation in further statistical analyses. Bradykinin-induced dilations were bimodal with peaks at 40% and 80% of maximum dilation. Therefore, dilation greater than 40% of maximum dilations was used as the primary endpoint in statistical analyses to predict the impact of other covariates on vessel function.

### Interactions between obesity, comorbidities and medications on coronary arteriole dilation

2-way ANOVA’s examining the interaction between obesity and co-morbid conditions, and between obesity and surgical procedures (coronary artery bypass vs. valve replacement) on coronary resistance vessel responses to bradykinin did not reveal any significant interactions (Table 2A). Two-way ANOVA examining the interaction between obesity and cardiovascular medications are presented in Table 2B. A significant interaction between obesity and ACE inhibitors on the vasodilator response of coronary arterioles to bradykinin was observed, F(1,60)=7.469, P=0.008. This interaction consists of an unchanged vessel response in obese patients taking ACE inhibitors (51.0% vs. 63.5% of max dilation, P=0.237), but a significant decrease in bradykinin-induced dilation of non-obese patients taking the ACE inhibitors (58.2% vs. 33.5% of max dilation, P=0.007) (Table 3). A significant interaction between obesity and beta-blockers on the response of coronary arterioles to bradykinin was also observed, F(1,60)=7.581, P=0.008. It should be noted, however, that nearly 90% of obese patients were taking beta-blockers at the time of surgery (Table 1) and thus the applicability of this test is severely limited. In non-obese patients, stratification by use of ARB’s and statins revealed greater dilations in those patients taking these medications. Stratification of the use of any medications within the obese population did not reveal any significant effects on arteriole responses.

**Table 2:** 2-Way ANOVAs assessing (A) the interaction between obesity and co-morbid conditions on coronary dilations and (B) the interaction between obesity and commonly prescribed medications on coronary dilations

**A**		
**Interaction with obesity**	**F test**	**P value**
Diabetes	0.043	0.836
Hypertension	0.587	0.447
Coronary artery disease	1.714	0.195
Hypercholesterolemia	3.634	0.061
Coronary artery bypass grafting	0.023	0.880
Aortic valve replacement	0.033	0.856
**B**		
**Interactions with obesity**	**F test**	**P value**
ACE - Inhibitor	7.469	0.008
Angiotensin receptor blocker	3.801	0.056
Aspirin	0.081	0.777
Statin	3.923	0.052
Insulin	2.196	0.144
Oral anti-diabetic drug	0.186	0.668
Beta blocker	7.581	0.008
Diuretic	1.513	0.224
Calcium channel blocker	0.904	0.345
H+ blocker	0.449	0.505
Nitrate	0.864	0.356

ACE – angiotensin converting enzyme inhibitor, Degrees of freedom and the error of degrees of freedom all have values of 1 and 60, respectively.

**Table 3:** Coronary dilations stratified by medications commonly prescribed to patients in obese or non-obese patients

	**BMI ≥ 30**			**BMI < 30**		
	**Bradykinin response [10-8M] -% dilation (Std. Error)**			**Bradykinin response [10-8M] -% dilation (Std. Error)**		
	**(+) Drug**	**(-) Drug**	**P Value**	**(+) Drug**	**(-) Drug**	**P Value**
**ACE inhibitor**	63.5 (9.5)	51.0 (5.3)	0.237	33.5 (6.2)	58.2 (5.8)	0.007
**Angiotensin receptor blocker**	55.1 (6.1)	54.1 (7.2)	0.919	64.4 (8.1)	36.9 (5.1)	0.007
**Aspirin**	57.1 (4.8)	43.1 (14.6)	0.261	49.2 (5.3)	30.5 (9.2)	0.086
**Statin**	58.3 (4.7)	50.4 (8.6)	0.412	53.7 (5.1)	20.1 (6.0)	0.001
**Insulin**	48.3 (7.6)	56.3 (5.6)	0.492	57.9 (10.3)	41.3 (5.2)	0.165
**Anti-diabetics**	62.0 (23.0)	52.6 (5.1)	0.424	46.2 (11.5)	44.1 (5.3)	0.867
**Beta blocker**	89.3% of obese patients are on beta blockers; test not valid			51.5 (5.7)	34.8 (7.6)	0.081
**Diuretic**	50.3 (9.8)	55.8 (5.4)	0.641	52.3 (6.0)	38.9 (6.7)	0.166
**Calcium channel blocker**	55.8 (7.6)	53.5 (6.1)	0.811	38.6 (7.2)	49.2 (6.2)	0.267
**H+ blocker**	62.0 (6.8)	49.8 (62.6)	0.210	45.8 (7.9)	44.3 (5.5)	0.907
**Nitrate**	59.9 (8.6)	52.5 (5.7)	0.487	11.1% of non-obese patients are on nitrates; test not valid		

BMI – body mass index; ACE – angiotensin converting enzyme; ARB – angiotensin receptor blocker; CCB – calcium channel blocker, Significance determined by 1-Way ANOVA. Data is presented as the mean ± standard deviation.

### Effects of obesity on adjusted models of coronary arteriole dilation to bradykinin

The multiple regression model evaluating the influence of obesity and medications on coronary arteriole responses to bradykinin (10^-8^ M) is presented in Table 4. Obesity and statin use were the only predictors of greater arteriole dilations to bradykinin. Multivariable logistic regression models predicting coronary vessel responses to bradykinin are presented in Table 5. The model predicts improved vessel dilations for obese patients, and for those taking statins. ACE inhibitors and ARBs, although significant in univariate models, did not demonstrate significant effect in the multivariable models. Inclusion of these medications did not alter the hazard ratios for statins and obesity compared to other models. Goodness of fit tests failed to reject the null hypothesis (P > 0.05) that the logistic model adequately represents the data.

**Table 4:** Multiple regression

**Covariate**	**B**	**Std. Error**	**Beta**	**t**	**Significance**	**R**^**2**^
Statins	0.21	0.07	0.38	3.16	0.002	0.17
Obesity	0.14	0.07	0.26	2.17	0.034	

**Table 5:** Multivariable logistic regression models examining the effects of obesity and medications on coronary arteriole dilations to bradykinin

**Covariate**	**Significance**	**HR**	**95% CI**	**HL Test**
BMI ≥ 30	0.03	5.00	1.18-21.13	0.27
Statin	< 0.01	7.98	1.76-36.19	
ARB	0.58	1.52	0.34-6.81	
ACE-I	0.95	1.04	0.28-3.86	

HR - hazard ratio; CI - confidence interval; HR - Hosmer and Lemeshow Test for goodness of fit; BMI – body mass index; ARB – angiotensin receptor blocker; ACE-I – angiotensin converting enzyme inhibitor.

Based on the outcomes of the multivariable models, which suggest a significant role for statins in predicting arteriole dilations to bradykinin, the influence of statin use within the whole population as well as between obese and non-obese populations was evaluated. We have found that statin therapy was associated with a significantly enhanced coronary dilation to bradykinin (Figure 1B). Furthermore, stratification of obese and non-obese patients by the use of statins demonstrated an enhanced dilation to bradykinin in non-obese patient taking statins and also in obese patients with or without statin medication, when compared to non-obese patients without statins (Figure 1C).

## Discussion

The novel findings of the present study are that both obesity and statins are independent predictors of enhanced dilation of coronary resistance arteries in patients undergoing heart surgery. The observed enhanced dilator function of coronary arterioles may independently contribute to improved myocardial perfusion during or after heart surgery, and may, in part, offer an explanation to the improved cardiovascular outcomes in these settings - the obesity paradox.

The obesity paradox was first described in 1999 by Fleishmann et al. who found that a one unit increase in BMI is associated with a 4% reduction in the relative risk of death in hemodialysis patients [16]. Since then, other patient populations have been identified where those with elevated BMI unexpectedly exhibit decreased morbidity and mortality in comparison to patients with normal BMI. These conditions include: acute and chronic heart failure, coronary artery disease, hypertension, percutaneous coronary intervention, stroke, intensive care unit patients, chronic obstructive pulmonary disease and chronic kidney disease [8-10,17].

The notion that obesity is being protective in patients who require heart surgery prompted us to raise the possibility that myocardial perfusion, hence coronary microcirculation, is less compromised in obese patients than is usually considered to be the case. In this context, earlier studies using positron emission tomography [5] and magnetic resonance imaging [7] showed unaltered myocardial perfusion in obese patients without coronary risk factors, while myocardial blood flow was found reduced only after metabolic or pharmacologic challenge [6]. Although such non-invasive imaging techniques are powerful tools to measure myocardial blood flow, conclusions are limited as these techniques provide only relative comparisons of myocardial perfusion and are unable to assess the microcirculation independently [18].

Herein we present the results of a study in which the dilator function of *ex vivo* coronary resistance arteries to the agonist, bradykinin was directly assessed. This study builds upon our previous observation that coronary arterioles isolated from obese patients exhibit enhanced dilations to the endothelium-dependent agonist, bradykinin and the NO donor, sodium nitroprusside in the setting of concomitant hypertension [11]. In the present study we set out to examine whether or not the observed effect of obesity on coronary responsiveness was influenced by the patients’ medications. Our sample size (64 consecutive patients) allowed us to employ robust statistical models to identify independent predictors of coronary arteriole dilation.

Importantly, our study identified obesity as an independent, positive predictor of coronary arteriole dilations, in patients undergoing heart surgery, with a 5.00 fold increase in the probability of dilating 40% or greater in response to the endothelium-dependent vasodilator, bradykinin (Table 4). Two-way ANOVA comparing the interactions between obesity and co-morbid conditions, and between obesity and medications, demonstrated no significance (Tables 2A and B). These data, together with the multivariable regression analysis, indicate that differences in coronary arterial dilations between obese and non-obese patients are due to obesity, rather than medications and other diseases. Given that, it is plausible that obesity is associated with an augmented coronary dilation, which may correlate enhanced myocardial perfusion in patients undergoing heart surgery. In this context, a recent retrospective analysis by van Straten et al. examining post CABG mortality in a large patient population (10,268 patients) showed that only morbid obesity (BMI > 35) was an independent predictor for late (> 30 days), but not early (< 30 days) mortality [19], whereas no significant effects on mortality rates were detected in overweight and obese patient populations. Intriguingly, in the unadjusted Kaplan-Meier survival curves, stratified by only preoperative BMI, the authors found a lower survival rate among patients who were normal weight when compared to patients who were overweight. Patient who were underweight had significantly lower survival compared with all other BMI groups [19]. The average BMI of obese patients in our study was 33.4 kg/m^2^. Of these patients only 5 were morbidly obese, and only 2 of the non-obese population were underweight. Given these small numbers, stratification of the data to include underweight and morbidly obese populations was not possible for analysis in the present study. Moreover, due to the nature of our study design – using unidentified surplus surgical specimens – the clinical outcome of heart surgery in obese versus non-obese patients was not investigated. Thus, further studies are needed in order to evaluate to what extent the level of adiposity affects coronary microvascular responses, which may also correlate with the short- and/or long-term outcome of patients undergoing heart surgery.

In this study we also found that statin therapy improves dilator function of coronary resistance arteries in patients undergoing heart surgery. This finding may be expected, but this study provides a direct evidence for the beneficial effects of statin therapy on vasodilator function in coronary resistance arterioles. When obese and non-obese patients were stratified depending on the use of statins, interestingly we found a significantly enhanced coronary dilation in obese subgroups, with our without statin, when responses were compared to non-obese patients with no statin treatment. Previous studies by Vaduganathan et al. found pre- and perioperative statin therapy to be associated with reduced mid-term mortality in patients undergoing heart surgery [20,21]. Recent randomized studies by Antoniades et al. demonstrated that pre-operative atorvastatin treatment (40 mg/day for three days) improved saphenous vein relaxations, effects that were independent from the lipid-lowering properties of the drug [22,23]. Our study offers further support and direct demonstration for the beneficial effect of statin therapy likely contributing to improved myocardial perfusion following heart surgery through enhancing the dilator function of coronary resistance arteries.

ACE inhibitors are also known to exhibit cardiovascular benefits [24] and have been shown to improve vascular function in animal models of obesity [25,26]. Although approximately half of the patients undergoing heart surgery are on ACE inhibitors, a controversy regarding their preferential use prior to CABG surgery exists [27]. In our present study ACE inhibitors taken prior to heart surgery had no significant effects on the magnitude of bradykinin-induced dilation in obese patents even though significant interactions between obesity and ACE inhibitors were identified. Our finding that non-obese patients who were taking ACE inhibitors had reduced coronary arteriole dilation is likely due to the disproportionate number of patients taking statins - 65% of non-obese patients on ACE inhibitors were taking statins, as compared to 81% of non-obese patients not on ACE inhibitors taking statins. Indeed, after controlling for the effects of statins in the multivariable model, it was observed that ACE inhibitors were not predictive of vessel dilations within our sample cohort. Moreover, it is also possible that ACE inhibitors, via increasing the tissue level of bradykinin may cause reduced receptor sensitivity to bradykinin, a potential mechanism, which may contribute to the observed reduced dilation of coronary arterioles, *ex vivo*; and also may explain the apparent controversy.

## Conclusion

In conclusion, our study demonstrates that obesity is independently associated with enhanced coronary arteriole dilation in patients undergoing heart surgery. Moreover, we identified statins as another important, independent predictor of dilation of coronary resistance arteries. Whether or not the enhanced arteriole dilation in obese patients, with or without statin therapy correlates with short- and longer-term outcomes following surgery has yet to be elucidated.

## Abbreviations

BMI: Body mass index; ACE: Angiotensin converting enzyme; ARB: Angiotensin receptor blocker; IHD: Ischemic heart disease; MRI: Magnetic resonance imaging; ANOVA: Analysis of variance; EC50: half maximal effective concentration.

## Competing interests

The authors declare that they have no competing interests.

## Authors’ contributions

JC, AF and ZB carried out the functional experiments. JC and LL performed the statistical analysis. JC, LL, VSP, DCA and ZB participated in the design of the study and wrote the manuscript. All authors read and approved the final manuscript.
